# Differential Resting-State Brain Characteristics of Skeleton Athletes and Non-Athletes: A Preliminary Resting-State fMRI Study

**DOI:** 10.3390/brainsci14101016

**Published:** 2024-10-12

**Authors:** Xinhong Jin, Shuying Chen, Yapeng Qi, Qichen Zhou, Jian Wang, Yingying Wang, Chenglin Zhou

**Affiliations:** 1 Key Laboratory of Exercise and Health Sciences, Shanghai University of Sport, Ministry of Education, Shanghai 200438, China; jinxinhong@sus.edu.cn; 2 School of Psychology, Shanghai University of Sport, Shanghai 200438, China; chenshuying0404@gmail.com (S.C.); yapwngqi@foxmail.com (Y.Q.); zqc_paperonly@126.com (Q.Z.); jwang6658@gmail.com (J.W.); wangyingying@sus.edu.cn (Y.W.); 3 Key Laboratory of Motor Cognitive Assessment and Regulation, Shanghai 200438, China

**Keywords:** resting-state fMRI, amplitude of low-frequency fluctuation, skeleton athlete, functional connectivity, neural plasticity

## Abstract

(1) Background: This study investigates the resting-state brain characteristics of skeleton athletes compared to healthy age-matched non-athletes, using resting-state fMRI to investigate long-term skeleton-training-related changes in the brain. (2) Methods: Eleven skeleton athletes and twenty-three matched novices with no prior experience with skeleton were recruited. Amplitude of low-frequency fluctuation (ALFF) and seed-based functional connectivity analyses were explored to investigate resting-state functional magnetic resonance imaging (rs-fMRI) data, aiming to elucidate differences in resting-state brain function between the two groups. (3) Results: Compared to the control group, skeleton athletes exhibited significantly higher ALFF in the left fusiform, left inferior temporal gyrus, right inferior frontal gyrus, left middle temporal gyrus, left and right insula, left Rolandic operculum, left inferior frontal gyrus, and left superior temporal gyrus. Skeleton athletes exhibit stronger functional connectivity in brain regions associated with cognitive and motor control (superior frontal gyrus, insula), as well as those related to reward learning (putamen), visual processing (precuneus), spatial cognition (inferior parietal), and emotional processing (amygdala), during resting-state brain function. (4) Conclusions: The study contributes to understanding how motor training history shapes skeleton athletes’ brains, which have distinct neural characteristics compared to the control population, indicating potential adaptations in brain function related to their specialized training and expertise in the sport.

## 1. Introduction

Long-term learning of motor skills has the potential to induce structural and functional plasticity in the brain [1,2,3,4]. Motor skill training refines movements through repeated practice and interactions with the environment, ultimately leading to effortless execution [5]. Studies suggest that individuals who engage in regular and varied training show structural and functional alterations in brain regions, such as the prefrontal cortex and hippocampus, which are linked to the duration and intensity of training [6,7,8,9]. Additionally, recent reviews have extensively evaluated the involvement of the cerebellum in the context of motor imagery, underscoring its critical role in motor and cognitive functions [10].

Neuroimaging techniques have unveiled brain structural or functional plasticity in various types of professional athletes, such as skilled golfers [11], world-class mountain climbers [12], racing-car drivers [13], badminton players [14], and world-class archers [15]. These studies show that continuous motor skill training can cause changes in both brain structure and function, indicating potential differences in these changes among various types of athletes. Skeleton, a high-speed Winter Olympic sport, entails athletes sliding headfirst down a bobsleigh track on a sled referred to as the ‘skeleton’. It involves a technical aspect where the competitor must run while bent over, ensuring that their sled remains straight within the ice grooves on the track [16]. This sport demands heightened cognitive functions such as spatial awareness, motor coordination, and risk assessment. Its distinctive features, characterized by a fusion of velocity, precision, and mental acuity, prompt the potential long-term impact on brain plasticity. As a result, skeleton emerges as a multifaceted sport requiring cognitive flexibility, possibly causing changes in the athlete’s brain over time. However, these changes have been rarely explored.

Resting-state fMRI is a method used to explore the natural, spontaneous shifts in the blood-oxygenation-level-dependent (BOLD) signal among distinct brain regions, allowing for the investigation of brain network activity when not engaged in specific tasks [3,4]. An effective approach to investigating the impact of expertise on whole-brain plasticity in the absence of a specific task is through resting-state functional connectivity (rs-FC). The hypothesis proposes that alterations in functional connections due to expertise could reveal which brain areas are consistently used during training, potentially showing a clear sign of expertise even during rest. Additionally, during rest periods, we can collect data about local brain activity using different techniques. One such method is the measurement of the amplitude of low-frequency Fluctuations (ALFF), which assesses the strength of a specific time course within a typical low-frequency range (for example, 0.01–0.08 Hz). Another method is fractional ALFF (fALFF), which indicates the ratio of the power spectrum of low-frequency signals to that of the entire frequency range (e.g., 0–0.25 Hz), thus minimizing sensitivity to physiological noise. Lastly, regional homogeneity (ReHo) calculates the level of synchronization among fMRI time courses within a specific brain region [17,18,19].

Previous study used ALFF intensity and the seed-based method to explore resting-state modifications due to expertise in ballroom dancers [17]. The results found that ballroom dancers exhibited increased ALFF in several brain regions including the left middle temporal gyrus, bilateral precentral gyrus, bilateral inferior frontal gyrus, left postcentral gyrus, left inferior temporal gyrus, right middle occipital gyrus, right superior temporal gyrus, and left middle frontal gyrus. Additionally, ballroom dancers showed lower ALFF in the left lingual gyrus and altered connectivity patterns between the inferior frontal gyrus and temporal and parietal regions. Another study explored the brain activity during rest in badminton players who had an average of nearly 10 years of training [14]. Athletes also showed reduced ALFF in the left superior parietal lobule, as well as alterations in the connectivity between the left superior parietal and frontal areas. A previous 4-week short-term motor skill training study [8] reported a notable rise in the strength of resting-state functional connectivity within the right postcentral gyrus and right supramarginal gyrus over 2 weeks, and noted a subsequent decrease in connectivity strength within these regions from week 2 to week 4.

With the rapid progress in fMRI technology, researchers in neuroscience and sport psychology are increasingly focused on studying the plasticity in the central nervous system among excellent athletes. Although the physical requirements of skeleton have been thoroughly examined, the investigation into its potential effects on brain adaptability is still an emerging area of research. Based on previous research on neural processes during sports training, we utilized the blood-oxygenation-level-dependent (BOLD) signals and resting-state functional connectivity (FC) analysis to decipher the patterns of functional communication between distant brain regions during rest. Understanding how the brain adapts to the challenges of skeleton is important for uncovering the potential long-term effects of the skeleton training on brain plasticity. It also sheds light on the fascinating connection between high-speed winter sports and cognitive adaptations.

## 2. Materials and Methods

### 2.1. Participants

Eleven professional skeleton athletes (mean age = 22.91 ± 0.52 years) were recruited from the national skeleton team, including 6 males. Additionally, twenty-three students from Shanghai University of Sport (mean age = 21.17 ± 2.18 years), including 8 males, were recruited as the control group. All participants were right-handed and had no history of neurological disorders. Participants gave informed consent before participation and were compensated for their involvement. This research was sanctioned by the ethics committee of Shanghai University of Sport (102772019RT011). Participants read and signed an informed consent form in accordance with the principles specified in the Declaration of Helsinki. Table 1 displays the demographic details of the participants. The skeleton athletes were recruited from national skeleton team who met the following criteria: (1) had at least 3 years of professional training experience; (2) practiced more than three days a week, with each session lasting at least 2 h, over the past 3 years; (3) participated in world skeleton competitions within the last 3 years. The control group consisted of students from Shanghai University of Sport who had no sports training experience. Specifically, they had never tried skeleton and had not seen anything related to it in the media before.

### 2.2. Data Acquisition

We collected all MRI data using a 3.0-T Siemens Trio Tim MR scanner (Siemens Magneton Prisma, Siemens Healthcare GmbH, Erlangen, Germany). Before the scan, we made sure that participants did not have any metal implants or psychiatric conditions like claustrophobia. Participants were instructed to lie down comfortably and keep their heads still throughout the scanning procedure.

We used a gradient echo EPI sequence to acquire the rs-fMRI datasets with the following settings: repetition time (TR) = 2000 ms, echo time (TE) = 30 ms, flip angle = 90°, field of view (FOV) = 220 mm × 220 mm, data matrix = 64 × 64, thickness = 3.5 mm, 33 transverse slices covering the whole brain, and 240 volumes acquired in 8 min. During the rs-fMRI scan, participants were told to keep their eyes closed but stay awake, and to relax their minds without thinking about anything in particular. Additionally, we obtained high-resolution structural brain images (1 mm^3^ isotropic) for each participant using a T1-weighted 3D fast spoiled gradient-recalled echo (FSPGR) sequence. The sequence parameters were TR/TE = 2530 ms/2.98 ms, inversion time (TI) = 1100 ms, slice thickness of 1 mm, flip angle of 7°, FOV of 256 mm × 256 mm, data matrix of 256 × 256, bandwidth (BW) of 190 Hz/pixel, and 192 sagittal slices acquired over approximately 10 min. Both the rs-fMRI data and 3D high-resolution brain structural images were collected in a single session for each participant.

### 2.3. Data Preprocessing

MRI data were preprocessed using dpabi [20]. All DICOM files were converted to NIfTI format. Structural images underwent segmentation into gray matter, white matter, and cerebrospinal fluid using the DARTEL method. Functional images were processed as follows: (1) corrected for slice timing, with the first 10 time points discarded for each subject; (2) motion correction, ensuring that all participants exhibited head motion of less than 2 mm translation and 2° rotation, with no exclusions; (3) co-registered with the structural data; (4) spatially normalized to the Montreal Neurological Institute (MNI) space T1 Template and resampled to a voxel size of 3 × 3 × 3 mm^3^; (5) covariates such as linear trend, white matter nuisance signals, cerebrospinal fluid BOLD signal, and Friston 24 head motion were regressed out; (6) spatial smoothing was applied using an isotropic Gaussian kernel with a full width at half maximum of 6 mm. The voxel-wise time series were adjusted and filtered between 0.01 and 0.08 Hz to remove low-frequency drift and high-frequency noise. The data used for ALFF calculation were not filtered, and the data used for ReHo calculation were not smoothed during preprocessing.

### 2.4. ALFF Analysis

After completing the data preprocessing steps, we obtained ALFF maps as follows. ALFF shows how much brain regions spontaneously change in activity, and ALFF analysis provides a way to evaluate the dynamic characteristics of voxel-wide BOLD signals in the low-frequency range (0.01–0.08 Hz) effectively. We transformed the processed image time series into frequency domain using fast Fourier transform (FFT) to obtain the power spectrum. Then, the square root of each frequency in the power spectrum was calculated. Finally, we calculated the ALFF values by averaging the values of each voxel within the frequency range of 0.01–0.08 Hz [21,22,23].

### 2.5. Seed-Based Functional Connectivity Analysis

After completing the data preprocessing, we applied a temporal band-pass filter (0.01–0.08 Hz) to reduce low-frequency drift and physiological high-frequency noise. Then, we chose 6 spherical regions (each with a 6 mm radius) based on the ALFF results as regions of interest (ROIs). We extracted the average BOLD signal intensity time series from these ROIs. Finally, we performed functional connectivity analysis by calculating the Pearson correlation coefficient between the ROIs and all voxels in the brain.

### 2.6. Statistical Analysis

We conducted two-sample *t*-tests on standard mean ALFF (mALFF) and functional connectivity maps to compare the differences in ALFF and functional connectivity between the skeleton group and the control group. We used false discovery rate (FDR) correction (*p* < 0.05) to correct for multiple comparisons.

## 3. Results

### 3.1. ALFF

**Figure 1:**
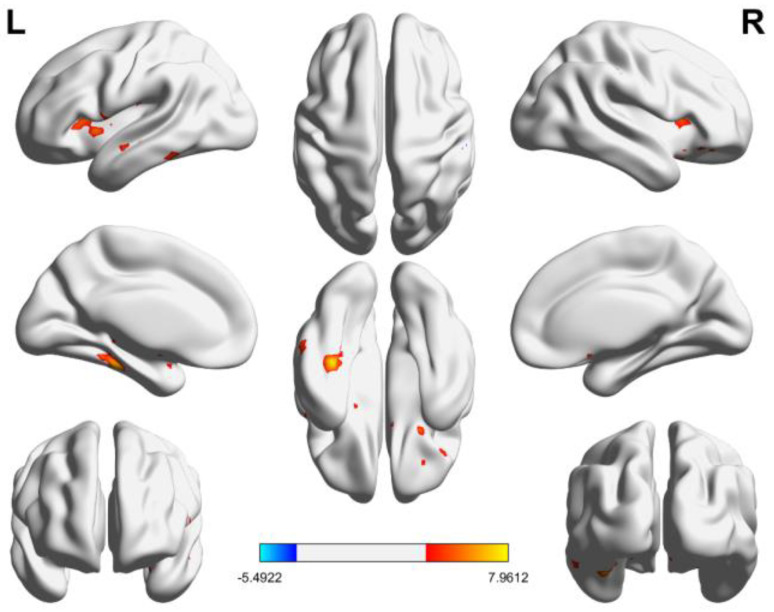
Inter-group comparison results of mALFF values. The color bar represents the *t*-values (*t* = 3.40). Warm colors indicate positive values (skeleton group minus control group), while cold colors represent negative values (skeleton group minus control group). Hemisphere designation: left (L) or right (R). Clusters with *p* < 0.05 and a spatial extent of *k* > 50 voxels were deemed statistically significant.

In comparison to the control group, skeleton athletes exhibited significantly higher mean ALFF (mALFF, Figure 1) in various brain regions including the left fusiform, left inferior temporal gyrus, right inferior frontal gyrus (orbital part), left middle temporal gyrus, left and right insula, left Rolandic operculum, left inferior frontal gyrus (opercular part), and left temporal pole (superior temporal gyrus, Table 2).

### 3.2. Functional Connectivity

**Figure 2:**
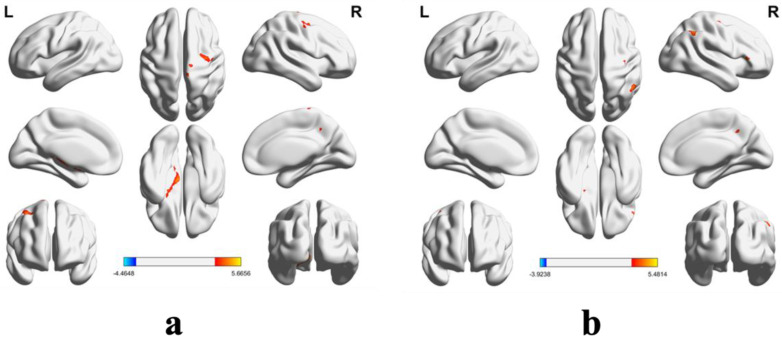
The inter-group comparison results of functional connectivity values. The functional connectivity results depicted in (**a**) are based on the seed region of interest located in the superior frontal gyrus. (**b**) displays the results utilizing the insula as the seed region of interest. The color bar indicates the *t*-values (*t* = 3.40). Warm colors indicate positive differences (skeleton group greater than control group), while cold colors represent negative differences (skeleton group less than control group) in either the left (L) or right (R) hemisphere. Clusters with *p* < 0.05 and a spatial extent *k* > 50 voxels were considered statistically significant.

Functional connectivity analysis revealed several differences between two groups (Table 3). Compared to the control group, skeleton athletes showed stronger connections from their superior frontal gyrus to various brain regions including the left putamen, left amygdala, right precuneus, right median cingulate and paracingulate gyri, right superior frontal gyrus (medial orbital), right precentral gyrus, right superior frontal gyrus (orbital part), and right paracentral lobule. Additionally, the insula of skeleton athletes exhibited stronger connections to the right inferior frontal gyrus (opercular part), right inferior frontal gyrus (triangular part), right middle temporal gyrus, right superior temporal gyrus, and right inferior parietal (supramarginal and angular gyri), as well as the right precuneus (Figure 2).

## 4. Discussion

Our current results reveal distinct activation patterns in various brain regions, indicating significant differences in both regional activity and functional connectivity between skeleton athletes and non-athletes. Firstly, elite skeleton athletes showed higher mean ALFF in the gray matter volume in the left fusiform, left inferior temporal gyrus, right inferior frontal gyrus (orbital part), left middle temporal gyrus, left and right insula, left Rolandic operculum, left inferior frontal gyrus (opercular part), and left temporal pole (superior temporal gyrus). Additionally, they showed stronger connectivity between the brain regions associated with cognitive and motor control (superior frontal gyrus, insula), as well as those related to reward learning (putamen), visual processing (precuneus), spatial cognition (Inferior parietal), and emotional processing (amygdala). These alterations could be attributed to the enhanced speed and coordination skills developed through prolonged skeleton training. Essentially, our findings support previous research suggesting that extensive practice of motor skills can lead to structural and functional changes in brain areas linked to motor control and execution.

Extended participation in skeleton training induces adaptive alterations across the cognitive and neural plasticity. The greater ALFF in the fusiform gyrus, frontal gyrus, temporal gyrus, and insula were consistent with the previous studies [7,17,19,24]. The left fusiform gyrus, found in the brain’s temporal lobe, is part of the ventral visual pathway. Its main role is in visual processing, especially in recognizing faces and objects [25]. This region is heavily involved in processing and representing visual information. It is reasonable to suggest that the increased activity in this area reflects the enhanced visual perception skills and cognitive demands of athletes developed through years of intense training. A recent study also discovered stronger connectivity between the posterior cerebellar lobe and fusiform gyrus in elite ice-skating athletes [19]. Additionally, it plays a crucial role in linking visual information with semantic meaning, allowing individuals to recognize and understand the significance of visual stimuli [26]. The inferior temporal gyrus helps athletes to swiftly recognize the track’s path, anticipate upcoming turns, and move through the course accurately. It also assists in spotting important landmarks or obstacles on the track, enabling athletes to swiftly adjust their movements. On the other hand, the middle temporal gyrus is associated with various cognitive functions, including language comprehension, auditory processing, and memory retrieval. It plays a role in memory formation and retrieval, aiding in the recollection of past events and experiences [27]. While not directly involved in the physical action of sliding down the track, this region assists athletes in comprehending verbal instructions from coaches, communicating well with teammates, and processing auditory feedback during training. It also helps in recalling memories of past runs and strategies, allowing athletes to learn from their experiences and improve their techniques over time. Moreover, engagement in the superior temporal gyrus has been linked to the synchronization of dance movements and correlates with the intensity of the movement coordination [28].

There is growing interest in studying the role of the right inferior frontal gyrus in executive control, particularly in tasks involving response inhibition and attentional control [29]. It helps skeleton athletes to quickly adapt and adjust their movements in response to changing conditions on the track. For instance, when unexpected obstacles or turns arise, athletes can hold back their first reactions and quickly decide how to move through the course well. This skill of holding back impulsive actions and staying concentrated is crucial for keeping control and accuracy while sliding down at high speeds. On the other hand, the left inferior frontal gyrus, known for its role in linguistic decision-making processes [30], can help athletes mentally practice their runs, visualize the track, and plan their approach to each turn and curve.

Many researchers propose that the insula is thought to play a role in motivating intentional movement by processing sensory signals from oneself and blending them with emotions and motivations [7,31]. Furthermore, both the left and right insula are involved in interoception, which is being aware of internal bodily states. This is especially important for skeleton athletes because they need to pay attention to their bodily sensations and movements during races to perform their best and stay safe on the track.

In the resting brain, there are natural low-frequency neuron activities that synchronize strongly over time among brain regions with similar functions. This synchronization during rest is called resting-state functional connectivity [3,7,24,26]. Researchers have observed precuneus activation during imagery tasks simulating walking, suggesting its involvement in effectively anticipating postural adjustments, coordinating movements, orienting spatially, and reacting to moving obstacles or individuals [32]. Based on a previous study on athletes [33], the precuneus emerges as a crucial brain region influenced by sports training. It is implicated in self-awareness, cognitive efficiency, and shifting attention [34,35], and it plays a key role in mentally visualizing movements for controlling body actions [36,37]. In the current study, we found that the precuneus has stronger connections with the superior frontal gyrus, which is linked to planning movements and controlling thinking. This suggests better coordination and efficiency in planning and carrying out movements. Also, its increased connections with the insula, which is involved in sensing internal body states and processing emotions, indicates better control over bodily reactions and emotions. This helps improve performance, especially under pressure. These enhanced connections also suggest potential targets for interventions such as cortico-cortical paired associative cortical stimulation (PACS) [38]. Strengthening these functional networks could improve rehabilitation outcomes in patients with neurological disorders, particularly in enhancing motor and cognitive functions post-stroke.

Existing research suggests that regular exercise promotes increased communication between different areas of the brain [7,13,18,23,33]; our findings reveal significant changes in brain connectivity linked to emotion regulation, cognitive control, motor coordination, reward processing, visual perception, spatial awareness, and memory among skeleton athletes. This indicates that participating in skeleton sports demands not just thinking and controlling movement as in other sports but also places unique demands on various cognitive and perceptual functions. These alterations in brain connectivity highlight the multidimensional nature of the cognitive and sensorimotor processes involved in skeleton racing. This shows how athletes have to manage feelings well, understand what they see, move around spaces, and learn from doing well while still controlling their movements precisely.

Despite the insightful findings of this study, it is important to acknowledge some of its limitations. First, the sample size may have impacted the generalizability of our findings. Although we aimed for a representative sample, a larger cohort could provide more robust results and enhance the statistical power of the analysis. Although the cerebellum was examined in this study, no significant differences were found. This may be due to limitations in sample size and experimental design, which could have affected the ability to detect relevant effects. Furthermore, the current study did not directly assess cognitive functions such as memory, attention, and executive functions in our subjects. Future studies could benefit significantly from integrating comprehensive cognitive assessments to enhance our understanding of how motor skill training influences both neural and cognitive outcomes. Future research could also further explore the practice of motor imagery among subjects to investigate potential correlations with the neuropsychological terms among athletes. Additionally, employing subject-specific analyses or higher-resolution imaging will enhance the ability to capture individual-level changes. These approaches would provide a more comprehensive understanding of the interplay between motor training and brain plasticity.

## 5. Conclusions

In comparison to the control group, skeleton athletes showed significantly increased ALFF in multiple brain regions, including the left fusiform gyrus, left inferior temporal gyrus, right inferior frontal gyrus, left middle temporal gyrus, left and right insula, left Rolandic operculum, left inferior frontal gyrus, and left superior temporal gyrus. Furthermore, skeleton athletes demonstrated enhanced functional connectivity in regions implicated in cognitive and motor control, as well as those related to reward learning, visual processing, spatial cognition, and emotional processing during resting-state brain activity. Present results add new evidence and may help us to understand the neural mechanisms of long-term skeleton skill training.

## Figures and Tables

**Table 1:** Demographic information.

	Skeleton Group	Control Group	*p* Value
Age (years)	22.91 ± 0.52	21.17 ± 2.18	>0.05
BMI (kg/m^2^)	23.82 ± 2.42	21.13 ± 3.15	>0.05
Years of training	4 ± 1.10	0	<0.01
Frequency (times/week)	11.27 ± 3.00	0	<0.01
Duration (hours)	2.14 ± 0.39	0	<0.01

Note: The demographic information of both the skeleton athletes and the control group was analyzed. There were no significant differences in age and BMI between the two groups (*p* > 0.05). However, significant differences were observed in terms of training years, frequency, and duration (*p* < 0.01). All skeleton athletes won placement in the World Championship or World Cup Championship. BMI = body mass index.

**Table 2:** Accuracy and reaction time under different information conditions (average ± standard deviation).

Brain Regions	H	MNI Coordinaties				Cluster Size	*t*-Value
		AAL	x	y	z		
Cluster1			−27	21	6	326	6.91
Insula	L	Insula_L				172	
Rolandic operculum	L	Rolandic_Oper_L				63	
Inferior frontal gyrus, opercular part	L	Frontal_Inf_Oper_L				22	
Temporal pole: superior temporal gyrus	L	Temporal_Pole_Sup_L				21	
Cluster2			45	15	−9	247	6.51
Insula	R	Insula_R				110	
Inferior frontal gyrus, orbital part	R	Frontal_Inf_Orb_R				71	
Cluster3			−36	−33	−24	99	7.96
Fusiform gyrus	L	Fusiform_L				67	
Inferior temporal gyrus	L	Temporal_Inf_L				25	
Cluster4			−54	−9	−12	57	4.92
Middle temporal gyrus	L	Temporal_Mid_L				51	

Note: The brain regions exhibiting significant differences in mALFF values are displayed below. The Automated Anatomical Labeling (AAL) atlas was utilized to identify the coordinates and sizes of these clusters within the brain regions. All clusters listed in the table surpass 20 voxels and underwent correction for multiple comparisons using false discovery rate (FDR), with a voxel-wise threshold of *p* < 0.05. Note: MNI: Montreal Neurological Institute; AAL: Automated Anatomical Labeling; ‘H’ denotes hemisphere, with ‘L’ representing left and ‘R’ representing right.

**Table 3:** Significant clusters in brain regions with resting-state functional connectivity between skeleton and control groups.

Seed ROIs	Cluster Location	MNI Coordinaties				Cluster Size	*t*-Value
		AAL	x	y	z		
Superior frontal gyrus, orbital part	cluster1		−24	−3	−9	257	5.67
	Putamen	putamen_L				31	
	Amygdala	Amygdala_L				21	
	cluster2		12	−54	45	153	4.92
	Precuneus	Precuneus_R				45	
	Median cingulate and paracingulate gyri	Cingulum_Mid_R				21	
	cluster3		27	42	−9	147	4.6
	Superior frontal gyrus, medial orbital	Frontal_Mid_R				20	
	cluster4		33	−12	57	111	4.68
	Precentral gyrus	Precentral_R				69	
	Superior frontal gyrus, orbital part	Frontal_Sup_R				23	
	cluster5		9	−18	78	61	4.96
	Paracentral lobule	Paracentral_Lobule_R				30	
Insula	cluster1		60	0	−3	176	5.48
	Inferior frontal gyrus, opercular part	Frontal_Inf_Oper_R				46	
	Inferior frontal gyrus, triangular part	Frontal_Inf_Tri_R				40	
	Middle temporal gyrus	Temporal_Mid_R				33	
	Superior temporal gyrus	Temporal_Sup_R				27	
	cluster2		54	−48	54	129	5.18
	Inferior parietal, but supramarginal and angular gyri	Parietal_Inf_R					
	cluster3		15	−51	39	99	4.96
	Precuneus	Precuneus_R				47	

Note: The seed regions of interest (ROIs) and the clusters exhibiting significant correlations with these seed ROIs are displayed. The Automated Anatomical Labeling (AAL) atlas was employed to identify the coordinates and sizes of these clusters within brain regions. All clusters listed in the table consist of more than 20 voxels and underwent correction for multiple comparisons using the false discovery rate (FDR) method, with a voxel-wise threshold of *p* < 0.05. MNI: Montreal Neurological Institute; AAL: Automated Anatomical Labeling; ‘L’ represents left and ‘R’ represents right.

## Data Availability

The data presented in this study are available on request from the corresponding author subject to privacy, ethical and legal clearance.
